# Trait variation across different life history stages of trees and its impact on net photosynthetic rate

**DOI:** 10.3389/fpls.2025.1657142

**Published:** 2025-11-05

**Authors:** Mingyuan Lu, Yuhan Song, Guangze Jin

**Affiliations:** ^1^ School of Ecology, Northeast Forestry University, Harbin, China; ^2^ Key Laboratory of Sustainable Forest Ecosystem Management of Ministry of Education, Northeast Forestry University, Harbin, China; ^3^ Northeast Asia Biodiversity Research Center, Northeast Forestry University, Harbin, China

**Keywords:** tree growth, plant functional traits, hydraulic traits, photosynthetic traits, life history stages

## Abstract

**Introduction:**

Exploring the variation in plant functional traits from different perspectives not only helps to reveal how plants adapt to their environment but also reflects their ecological strategies. This study investigated the differences in trait variability across different life history stages and how these differences affect the net photosynthetic rate of trees.

**Methods:**

The research measured photosynthetic traits, hydraulic traits, leaf morphological traits, and leaf stoichiometry at various life history stages, exploring the variation and coordination among functional traits at different stages.

**Results:**

Results showed that (1) the sapwood specific hydraulic conductivity and leaf specific hydraulic conductivity exhibited the highest variability among all traits, while carbon and phosphorus content had the lowest variability. (2) Intraspecific trait variation accounted for a significant portion of the total variance, indicating extensive plasticity in ecological strategies and environmental adaptability among individuals of the same species. (3) Regarding different life history stages, small trees surpassed mature trees in several physiological indicators, including higher leaf specific hydraulic conductivity, sapwood specific hydraulic conductivity, whole-branch hydraulic conductivity, and contents of carbon, nitrogen, and phosphorus. (4) As trees grew, both net photosynthetic rate and hydraulic efficiency tended to decline, with a weakened synergy between them, suggesting that water and nutrient transport efficiency are key factors limiting tree growth.

**Discussion:**

In summary, our findings emphasize the importance of water transport efficiency in photosynthesis and reveal the coordinated relationship between water transport and net photosynthetic efficiency across different life history stages of trees. These findings provide new insights into how trees adjust their functional traits to respond to environmental stress during their growth and have important implications for maintaining productivity and balancing biodiversity in forest ecosystems.

## Introduction

Plant functional traits refer to morphological or physiological features that significantly impact plant adaptability (Violle et al., 2007). Due to evolutionary and biophysical constraints as well as trade-offs, many functional traits are interrelated, forming groups of traits that vary together along key dimensions of plant strategy, a pattern consistently supported by global-scale analyses (Díaz et al., 2016, 2022; Joswig et al., 2022; Reich, 2014).For instance, water transport efficiency is highly coordinated with plant photosynthesis, and higher hydraulic conductivity is crucial for supporting high gas exchange rates and growth rates in plants (Gong et al., 2020). Therefore, due to pervasive phenotypic integration, changes in a single functional trait can have cascading effects on plant function by directly or indirectly altering the expression and influence of correlated traits (Maynard et al., 2022; Laughlin et al., 2020). The plasticity of plant functional traits can enhance a plant’s ability to respond to local environmental changes. Changes in plant morphology and internal physiology help reduce adverse environmental impacts (Song et al., 2012). Species with higher trait variability exhibit stronger trait-environment matching capabilities compared to those with lower variability (Mitchell et al., 2016). This trait variation includes species trait plasticity, enhancing their rapid response capability to environmental changes (Fox et al., 2019) and genotype adaptation to environmental changes over longer time spans (Murren et al., 2015). Although plant functional traits are fundamentally shaped by abiotic filters such as water availability, temperature, and soil nutrients (Gutschick and BassiriRad, 2003), they are equally forged by biotic interactions including competition, herbivory, and mutualism (Grime, 2006; McGill et al., 2006). Contemporary research emphasizes that these forces do not act in isolation but interact along environmental gradients to determine global patterns of trait variation and community assembly (Bruelheide et al., 2018). Therefore, considering the basic mechanisms driving variations driven by different factors is crucial for understanding and accurately modeling vegetation dynamics (Franklin et al., 2020).

Under environmental changes, a common theme in plant physiological responses is the trade-off between environmental stress tolerance and growth, that is, the safety and efficiency trade-off between leaf water coordination and photosynthesis (Blackman et al., 2016; de la Riva et al., 2016; Li et al., 2015; O’Brien et al., 2017). The relationship between water transport and photosynthesis reflects the trade-off between carbon uptake and water loss. The well-established coordination among leaf nutrients, hydraulic capacity, and photosynthetic rate means that leaves with high nutrient supply typically exhibit a more efficient water system and higher carbon assimilation (Niinemets, 2023; Brodribb et al., 2007).Moreover, leaf hydraulic conductance plays a particularly important role in the process of water transmission, because the entire plant’s water transport pathway forms a bottleneck at the leaf (Sack and Frole, 2006; Brodribb et al., 2007), and shares a common pathway through stomatal aperture for water conduction and leaf carbon dioxide exchange (Brodribb et al., 2007; Fiorin et al., 2016). Both water transpiration costs and carbon assimilation benefits occur within the leaf (McKown et al., 2010; Brodribb and Jordan, 2011; Simonin et al., 2012). Evidence robustly confirms that gas exchange and hydraulic conductivity are intrinsically coordinated across diverse tree species (Brodribb and Feild, 2000; Meinzer, 2002), a coupling now explained by mechanistic models of stomatal regulation and hydraulic limitation (Klein, 2014). Consequently, key leaf photosynthetic characteristics can be predicted by plant hydraulic traits, as the efficiency of water transport fundamentally constrains the capacity for carbon assimilation (Santiago et al., 2004; Li et al., 2020).Therefore, we can infer that hydraulic properties strongly affect photosynthetic rate, which affects the speed of water delivery to the leaves, while photosynthesis is limited by hydraulic properties because the water transported through the xylem must replenish the water lost through stomata during CO_2_ absorption (Brodribb, 2009). Many empirical studies (Brodribb et al., 2007; Scoffoni et al., 2016; Zhu et al., 2018) and optimization arguments (The optimization model) (Deans et al., 2020) support the close coordination between hydraulic and photosynthetic traits.

Tree size variation is one of the main axes of global plant morphology and functional trait spectra (Díaz et al., 2016). Among different tree species, variations in tree size have significant impacts on metabolism, structure, and function (Thomas, 1996; Brown et al., 2004). In taller trees, leaf physiological characteristics are affected by vertical gradients in light and water (Ambrose et al., 2009; Woodruff et al., 2009; Coble and Cavaleri, 2015; Chin and Sillett, 2017). Additionally, increases in gravity and transpiration path length increase water flow resistance, thereby affecting hydraulic conditions (Ryan et al., 2006), reducing stomatal conductance, and subsequently impacting photosynthesis (Ryan and Yoder, 1997; Barnard and Ryan, 2003). Studies have shown that hydraulic limitations are considered a key factor leading to the decline in physiological functions of tall trees (Kolb and Stone, 2000; Phillips et al., 2003; Koch et al., 2004; Ryan et al., 2006). Taller trees experience stronger hydraulic constraints because as height increases, so does evaporative demand and limitations in vertical water transport may restrict the physiological functions of leaves at the top of the canopy (Ishii et al., 2014). Therefore, tree size can affect physiological traits (Nabeshima and Hiura, 2008). Globally, larger trees suffer greater losses during drought periods due to being more susceptible to hydraulic stress (Bennett et al., 2015). Thus, elucidating differences in water transport efficiency among various tree sizes may help us understand changes in photosynthetic rates as trees grow in size.

Tree size has a significant impact on growth and adaptability, especially in terms of water transport and photosynthesis. Understanding how traits exhibit across different life-history stages (saplings, juvenile trees, and adult trees) is critical to revealing the mechanisms underlying the dominance of specific tree species in forest ecosystems. Moreover, identifying which functional traits exhibit variability between different life history stages and how they regulate net photosynthetic rate will help us understand the limiting factors of tree growth. Based on the aforementioned background, we formulated the following hypotheses: (1) Hydraulic traits would exhibit greater intraspecific variability and be more strongly influenced by life history stage than leaf morphological and chemical traits, reflecting their high sensitivity to ontogenetic changes in hydraulic architecture and light environment. (2) The relative importance of hydraulic traits in determining net photosynthetic rate (Pn) would surpass that of leaf nutrients, and this dominance would be most pronounced in adult trees due to heightened hydraulic constraints. (3) The tight coordination between hydraulic efficiency and photosynthesis would decouple as trees mature, providing a mechanistic explanation for the observed decline in photosynthetic capacity with tree size. To test these hypotheses, we measured a suite of functional traits across sapling, juvenile, and adult stages of six temperate broadleaf species. By deeply analyzing the above questions, this study hopes to provide scientific basis for understanding tree growth strategies in forest ecosystems and promote our understanding of global plant diversity and its ecological functions.

## Materials and methods

### Study sites

The field investigation was conducted in the Liangshui National Nature Reserve, China (47°10′50″ N, 128°53′20″ E), located in a temperate continental monsoon climate zone with an elevation ranging from 280 to 707 meters. This region experiences distinct seasonal changes, with short spring and autumn seasons often accompanied by strong winds, a brief and humid summer, and a long and cold winter. The average annual temperature is -0.3 °C, with an annual precipitation of approximately 676 mm, an evaporation rate reaching 805 mm, and a snow cover period lasting between 130 to 150 days.

The zonal vegetation within the reserve is predominantly composed of broadleaf Korean pine (*Pinus koraiensis*) forests, featuring undulating mountainous terrain. The main broad-leaved tree species include *Betula costata*, *Fraxinus mandshurica*, *Ulmus laciniata*, *Ulmus davidiana* var. *japonica*, *Acer pictum* subsp. *mono*, *Betula platyphylla*, *Phellodendron amurense*, *Acer ukurunduense*, and *Acer tegmentosum*, etc (Xu and Jin, 2012). These species form a unique forest ecosystem in the area, providing a rich biodiversity background for our research.

### Experimental design

**Figure 1:**
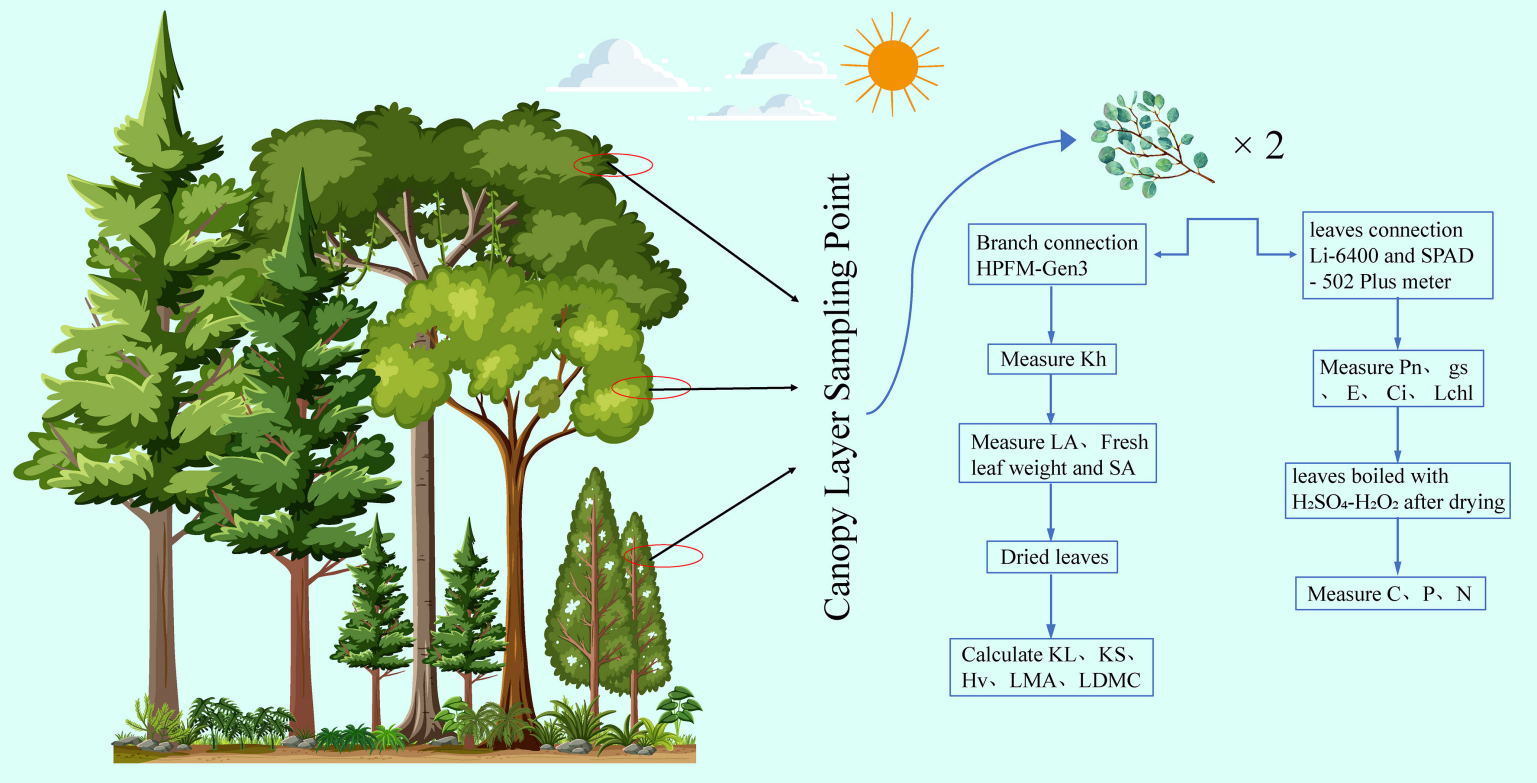
Schematic diagram of the experimental process illustrating the key parameters measured: KS (sapwood specific hydraulic conductivity), Kh (whole-branch hydraulic conductivity), KL (leaf specific hydraulic conductivity), E (transpiration rate), gs (stomatal conductance), Hv (Huber’s value), Pn (net photosynthetic rate), LMA (leaf mass per area), N (nitrogen content), LDMC (leaf dry matter content), Ci (intercellular carbon dioxide concentration), Lchl (chlorophyll content index), P (phosphorus content), C (carbon content), LA (total leaf area), and SA (cross-sectional area of the sapwood segment). The same abbreviations apply to subsequent figures.

At the sampling site, we selected six major broad-leaved tree species: *Acer tegmentosu*, *Fraxinus mandshurica*, *Juglans mandshurica*, *Acer pictum* subsp*. mono*, *Betula platyphylla*, and *Ulmus laciniata*. Based on the diameter at breast height (DBH), height, and within-canopy position, tree species were classified into three distinct size categories (adult trees, juvenile trees, saplings). In August 2021, a total of 90 trees were randomly selected as samples, with 5 trees per species. To ensure the representativeness and consistency of sampling position of the samples, all were collected from the uppermost portion of the sun-exposed side of the trees (**Figure 1**; **Table 1**). Samples for all measured traits were collected from current-year shoots. The shoot samples from adult trees were fully exposed to sunlight; those from juvenile trees were partially shaded; and those from saplings were fully shaded under the forest canopy. We acknowledge that the sample size per species and life stage (n=5) is modest relative to the number of traits analyzed. However, our experimental design focused on six ecologically important species, yielding a total of 90 individual trees. This replication at the community level, combined with the use of mixed-effects models that account for random effects of species and individual, provides robust statistical power to detect the major patterns of trait variation and their relationships across life history stages.

**Table 1:** Basic characteristics of the 6 tree species in this study.

Life history stages and light conditions	Sapling (under the forest canopy)		Juvenile tree (partially shaded)		Adult tree (exposed to sunlight)	
Species	DBH (cm)	H (m)	DBH (cm)	H (m)	DBH (cm)	H (m)
*Acer pictum* subsp*. mono*	2.78 ± 0.53	3.9 ± 1.2	17.56 ± 2.31	13.84 ± 2.06	30.64 ± 2.3	16.7 ± 4.5
*Betula platyphylla*	3.96 ± 0.48	5.76 ± 1.08	20.26 ± 1.17	18.94 ± 3.1	34.88 ± 3	22.14 ± 2.7
*Ulmus laciniata*	2.54 ± 0.42	3.58 ± 0.72	19.9 ± 1.15	15.52 ± 1.84	36.48 ± 2.94	22.54 ± 4.1
*Acer tegmentosum*	3.52 ± 0.42	3.88 ± 0.54	8.8 ± 0.32	9.22 ± 2.01	17.12 ± 0.4	15.38 ± 1.75
*Fraxinus mandschurica*	3 ± 0.45	4.02 ± 0.78	23.62 ± 0.95	24.12 ± 3.17	39.22 ± 3.81	27.46 ± 3.28
*Juglans mandshurica*	3.94 ± 0.95	4.84 ± 1.03	21.98 ± 1.51	23.1 ± 2.83	36.8 ± 1.66	28.36 ± 4.32

### Measurement of hydraulic traits, leaf traits, and photosynthetic traits

Sampling operations were conducted during the noon period, specifically between 12:30 and 13:00. To obtain the required samples from the upper canopy branches, we used high-branch pruners and manual climbing methods. From each selected sample tree, we cut approximately 50 cm long branches and promptly placed them in a bucket of water. Underwater, about 10 cm from the base of the branch was cut to prevent water loss. The upper end of the branch was then covered with a black plastic bag to further prevent water evaporation and immediately transported back to the laboratory. In the laboratory, we cut about 10 cm long sections of the branch in distilled water and peeled off about 1 cm of bark from the cut end. Then, the branches were connected to the pressure coupler of a high-pressure flow meter (HPFM-Gen3, Dynamax, Houston, USA). The instrument was set to a quasi-steady state measurement mode, and degassed distilled water was injected into the branches at a stable pressure of 0.42 MPa until a steady flow rate was reached (approximately 15 to 25 minutes), thereby measuring the whole-branch hydraulic conductivity (Kh, mg s^-1^ MPa^-1^). Subsequently, we scanned the leaves using a scanner (LiDE120, Canon, China) and used ImageJ software to obtain the total leaf area (LA, m^2^) at the end of each branch. The cross-sectional area of the sapwood segment (SA, m^2^) was also measured. The calculations for leaf specific hydraulic conductivity (KL, mg m^-1^ s^-1^ MPa^-1^) and sapwood specific hydraulic conductivity (KS, mg m^-1^ s^-1^ MPa^-1^) were based on the following formulas: KL = (Kh × 0.1m)/LA and KS = (Kh × 0.1m)/SA. The Huber value (Hv) was calculated using the formula Hv = SA/LA.

The leaves from the branches used for hydraulic conductivity measurements were weighed for fresh mass using an electronic balance (AR2140, New Jersey, USA) with a precision of 0.0001 g. Subsequently, the leaves were placed in an oven at 65°C to dry for 48 hours to determine their dry mass. From these data, we calculated the leaf mass per area (LMA, g/m²) and leaf dry matter content (LDMC). The LMA was obtained by dividing the leaf area by the leaf dry mass, reflecting the mass of the leaf per unit area; LDMC was determined by dividing the leaf dry mass by the leaf fresh weight, which is an important parameter indicating the proportion of dry matter in the leaf and reflects the plant’s water strategy.

To measure the photosynthetic rate of the sample trees, mature leaves were collected from the sunlit side of each tree. Measurements were taken using a Li-6400 (LI-COR, Lincoln, USA) photosynthesis system, with the light intensity set at 1500 μmol·m^−2^;·s^−1^ and CO_2_ concentration at 400 μmol mol^−1^. According to previous experience, under these conditions, the leaves can achieve the maximum net photosynthetic rate (Mo et al., 2019). On each tree, three leaves were selected to measure the maximum net photosynthetic rate based on leaf area (Pn, μmol m^−2^;·s^−1^), stomatal conductance (gs, μmol m^−2^;·s^−1^), transpiration rate (E, mmol H_2_O m^−2^;·s^−1^), and intercellular carbon dioxide concentration (Ci, μmol CO_2_ mol^−1^). All photosynthetic parameters were measured under field conditions to ensure the ecological relevance and physiological accuracy of the results. After completing the photosynthetic measurements, the chlorophyll content index (Lchl) of the leaves was measured using a SPAD-502 Plus meter (Konica Minolta, Tokyo, Japan), avoiding the main veins of the leaves. The leaves used for photosynthetic measurements were kept fresh, while the remaining leaves were dried in an oven at 65°C to constant weight for subsequent chemical analysis. Dried and ground samples were sieved through a 100-mesh screen and then digested using an H_2_SO_4_-H_2_O_2_ method to prepare them for the determination of carbon (C, mg/g), nitrogen (N, mg/g), and phosphorus (P, mg/g) contents. A multiN/C 3000 analyzer and a CleverChem 380 automatic discrete analyzer were used to measure these element contents.

### Data processing and analysis

To thoroughly assess the variability of traits, we calculated the interquartile coefficient of dispersion (QCD) for each trait. This index is obtained by taking the ratio of the half-interquartile range [i.e., the difference between the third quartile (Q3) and the first quartile (Q1)] to the average of the quartiles (i.e., (Q1+Q3)/2). This coefficient allows us to quantify the degree of dispersion of the traits. To understand the distribution of trait variability at different levels, we employed a fully nested ANOVA with nesting levels: species > life history stages > individuals. This approach enabled us to evaluate how trait variability is distributed across these different levels. Principal component analysis (PCA) was used to explore the overall covariation and trade-offs among functional traits across different life history stages. Linear mixed models were used to assess the impact of various functional traits (including hydraulic traits, leaf photosynthetic traits, leaf morphological traits, and leaf stoichiometry) on net photosynthetic efficiency. Structural equation modeling (SEM) was constructed to study the effects of tree size and different traits on the net photosynthetic rate. SEM allows us to consider multiple variables simultaneously and reveal the underlying structures behind these relationships. Finally, multiple linear regression was used to analyze the effects of sapwood specific hydraulic conductivity and leaf area-specific hydraulic conductivity on the net photosynthetic rate.

For statistical analysis, Minitab 19 was used for the fully nested ANOVA, while various packages in R 4.2.3 (R Development Core Team), including lme4 (Bates et al., 2015) for linear mixed models, piecewiseSEM (Lefcheck, 2016) for structural equation modeling, FactoMineR (Husson et al., 2017) for principal component analysis, and ggplot2 (Wickham, 2016) for linear regression plotting, were utilized. To ensure data normality, all data underwent log transformation.

## Results

### Variation and distribution of tree functional traits across different life history stages

**Figure 2:**
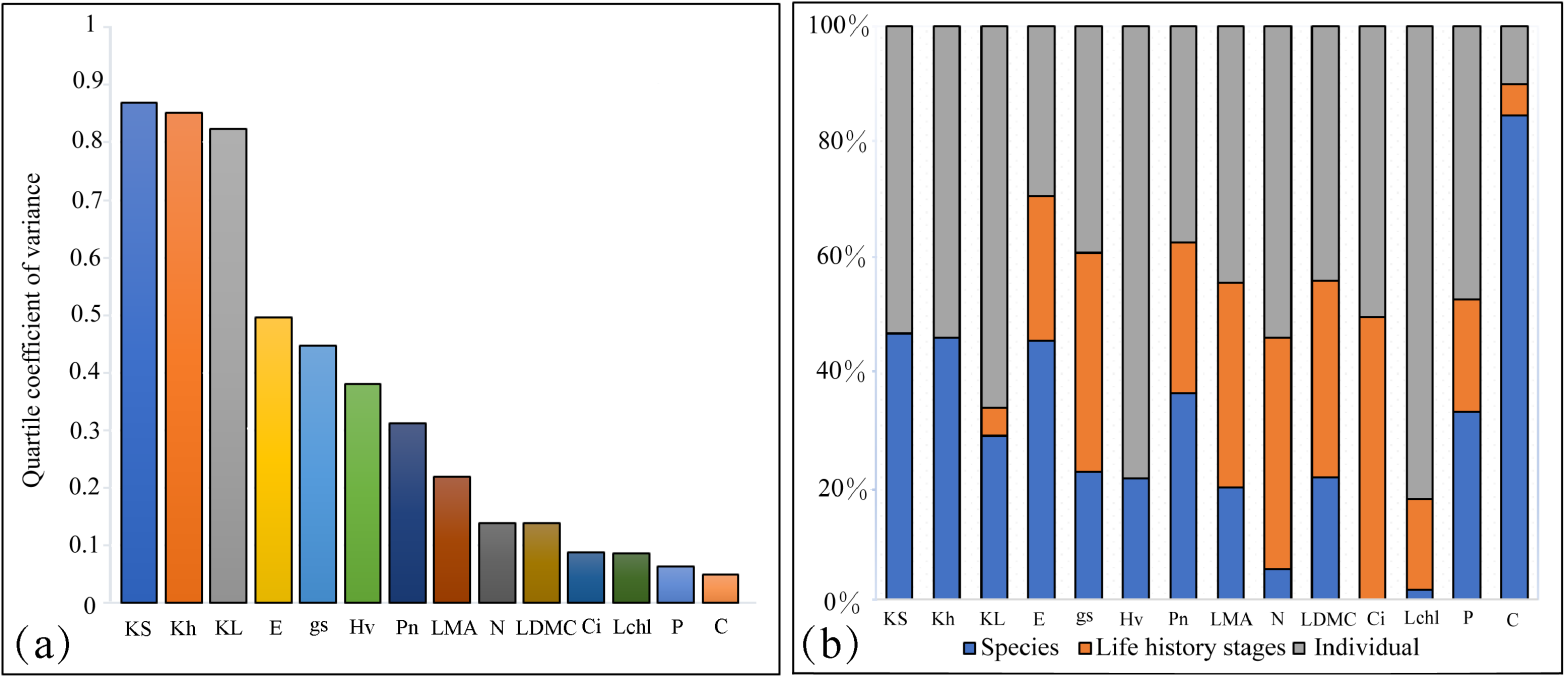
Variation and variance decomposition of tree functional traits across different life history stages. **(a)** Variation in different functional traits. **(b)** Variance decomposition of different functional traits. Percentages indicate the proportion of variance explained by each factor; values below 0.1% are displayed as 0. Trait abbreviations are consistent with those defined in **Figure 1**.

Trait variation was primarily observed within and between species, with species differences explaining an average of 29.26% of the variance in tree functional traits (**Figure 2**). This was particularly evident for traits related to water transport, such as sapwood specific hydraulic conductivity, whole-branch hydraulic conductivity, transpiration rate, net photosynthetic rate, and carbon content, where species differences accounted for more than 40.00% of the variance. This indicates a significant role of interspecific differences in these traits. In contrast, life history stages had a smaller impact on the variation of tree functional traits, with an average explanation of 21.08%. However, for certain leaf traits like stomatal conductance, nitrogen content, intercellular carbon dioxide concentration, leaf mass per area, and leaf dry matter content, the explanatory power of life history stages was higher, exceeding 30.00%. When considering trait variability, hydraulic traits such as whole-branch hydraulic conductivity, sapwood specific hydraulic conductivity, leaf specific hydraulic conductivity, Huber’s value, and transpiration rate showed greater variability. In contrast, traits like carbon content, phosphorus content, and chlorophyll content index exhibited relatively lower variability (**Figure 2**).

**Figure 3:**
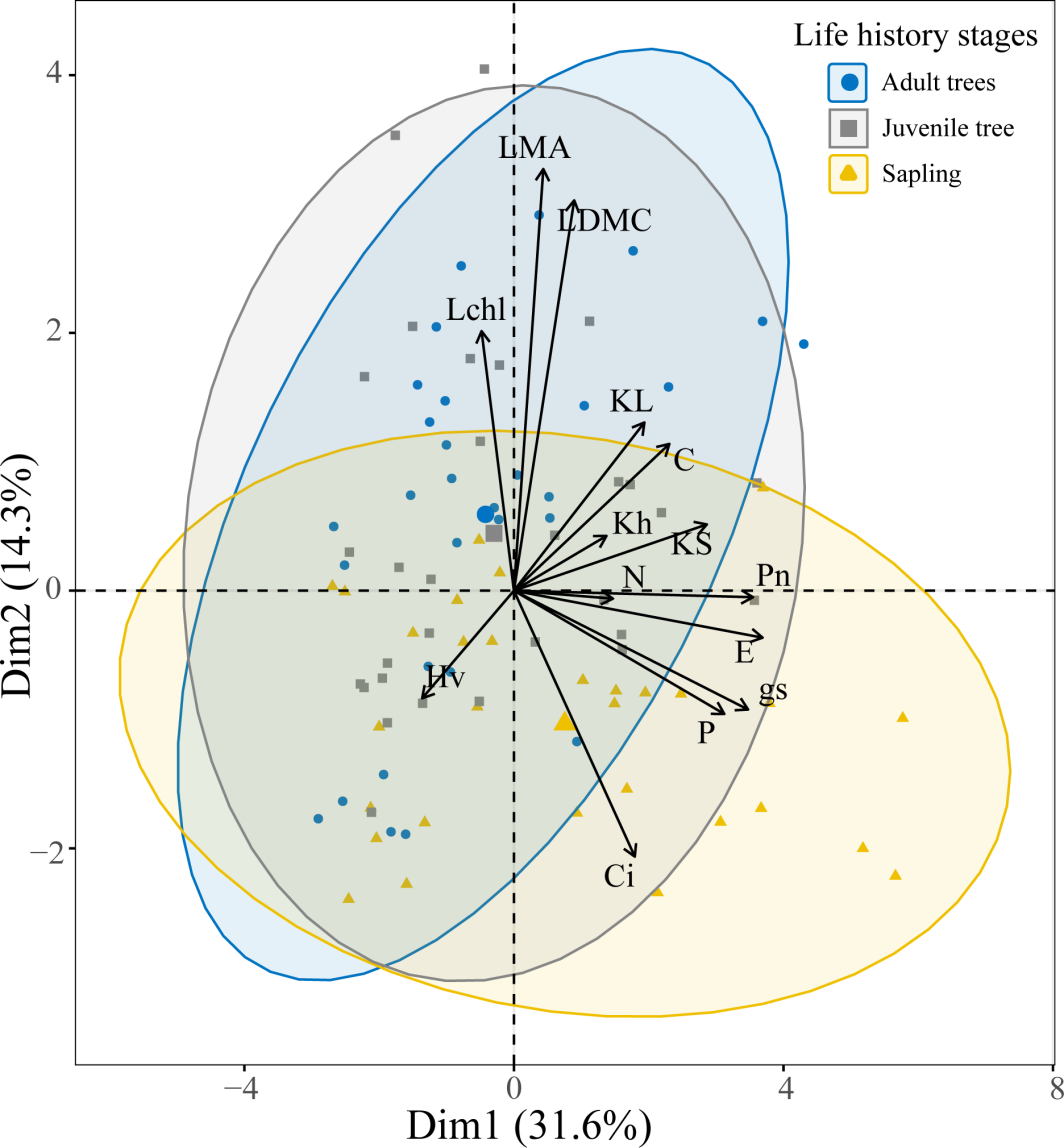
Principal component analysis of tree functional traits across different life history stages. Traits are abbreviated as detailed in **Figure 1**.

The results of the principal component analysis (PCA) indicate that the first two principal components (PCs) account for 45.9% of the total variance, meaning these two PCs can explain nearly half of the variation in the 14 functional traits. The first principal component primarily encompasses traits related to water transport and nutrient content, including leaf specific hydraulic conductivity, sapwood specific hydraulic conductivity, whole-branch hydraulic conductivity, carbon content, nitrogen content, phosphorus content, net photosynthetic rate, stomatal conductance, and transpiration rate. The second principal component mainly includes traits associated with leaf structure and photosynthesis, such as Huber’s value, leaf dry matter content, leaf mass per area, intercellular carbon dioxide concentration, and chlorophyll content index. In trees across different life history stages, saplings are more distributed on the positive side of the first principal component, indicating that they tend to have higher values in traits such as leaf specific hydraulic conductivity, sapwood specific hydraulic conductivity, whole-branch hydraulic conductivity, carbon content, nitrogen content, phosphorus content, net photosynthetic rate, stomatal conductance, and transpiration rate. In contrast, juvenile and adult trees are more distributed on the positive side of the second principal component, which may reflect their higher values in traits like leaf dry matter content, leaf mass per area, and chlorophyll content index (**Figure 3**).

### Contribution and relative importance of predictors for net photosynthetic efficiency

**Figure 4:**
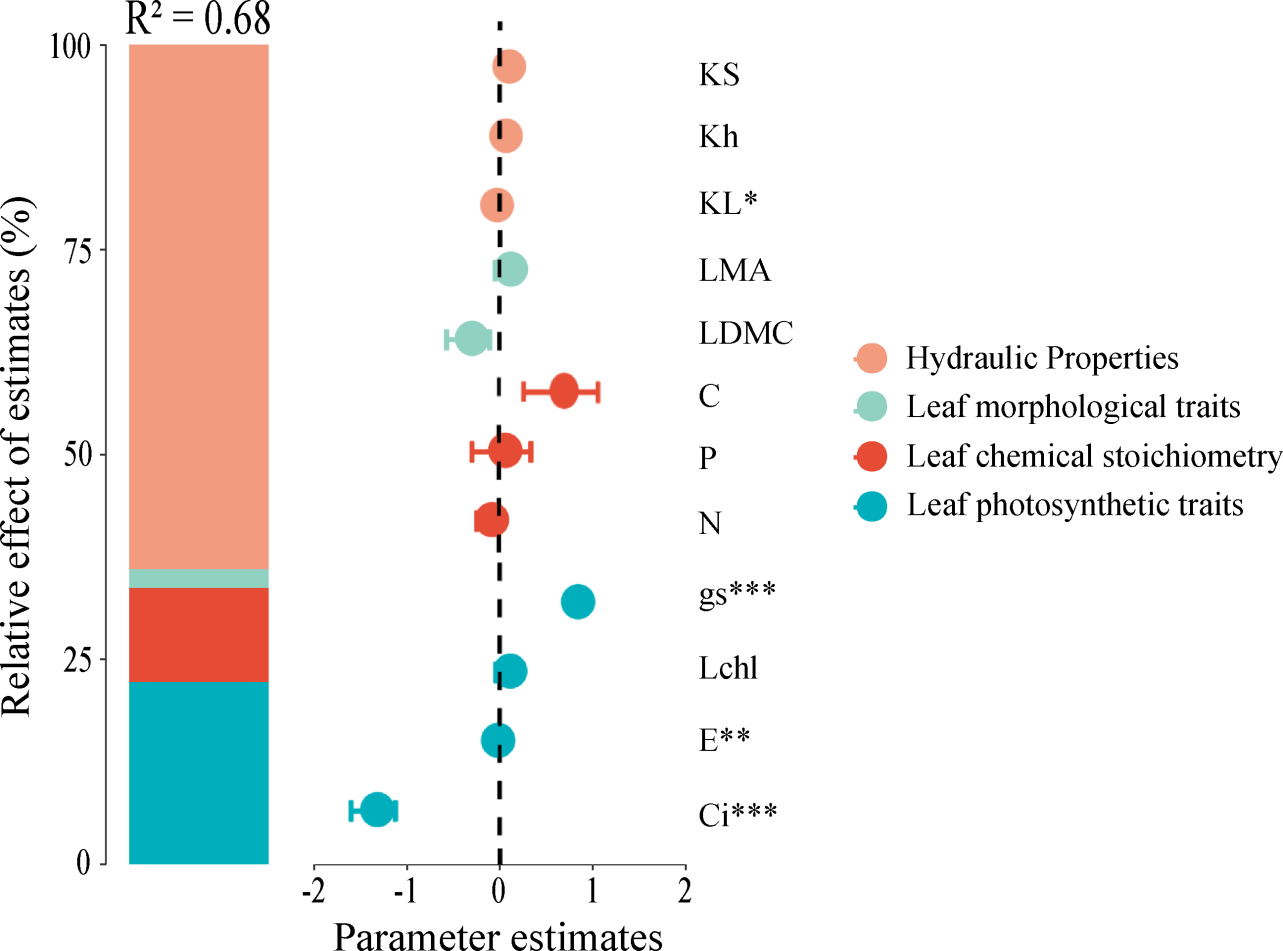
Relative importance of functional traits in predicting net photosynthetic rate (Pn). Significance levels are denoted by asterisks: *P < 0.05, **P < 0.01, ***P < 0.001. Trait abbreviations follow **Figure 1**.

A mixed-effects model was employed to analyze the relative importance of hydraulic traits, leaf morphological traits, leaf chemical traits, and leaf chemical stoichiometry in predicting net photosynthetic efficiency. The model incorporated all predictors and demonstrated high explanatory power with a coefficient of determination (R²) of 0.68, indicating that the predictors within the model adequately explain the variance in the dependent variable. The results revealed that hydraulic traits, leaf morphological traits, leaf chemical stoichiometry, and leaf photosynthetic traits accounted for 59.28%, 0.27%, 15.92%, and 24.53% of the variation in net photosynthetic rate, respectively. This finding highlights that hydraulic traits are the primary factors influencing net photosynthetic rate, while leaf morphological traits have a minimal impact (**Figure 4**).

Within the model, traits such as stomatal conductance, transpiration rate, intercellular carbon dioxide concentration, and leaf specific hydraulic conductivity were significantly correlated with net photosynthetic rate. These traits play crucial roles in the photosynthetic process of plants, where stomatal conductance and transpiration rate are directly involved in gas exchange and water regulation, whereas intercellular carbon dioxide concentration and leaf specific hydraulic conductivity are closely linked to the efficiency of photosynthesis (**Figure 4**).

### Relationship between tree functional traits and photosynthetic rate across life history stages

**Figure 5:**
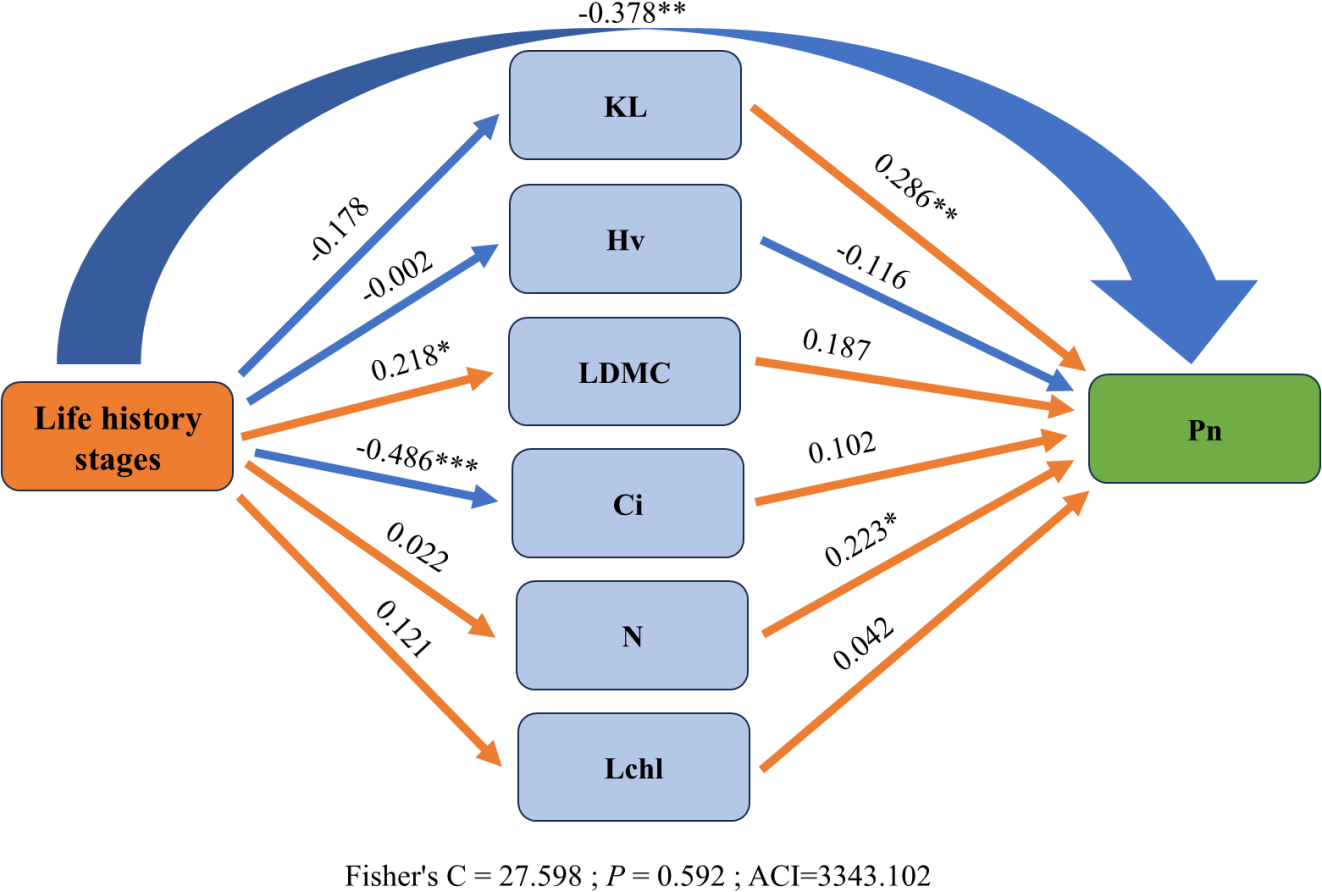
Relationships between functional traits and net photosynthetic rate (Pn) across life history stages. Orange and blue lines represent positive and negative correlations, respectively. Significance levels are indicated as *P < 0.05, **P < 0.01, ***P < 0.001. Abbreviations are consistent with **Figure 1**.

In the structural equation model (SEM), to examine the impact of different life history stages, trees were assigned values from 1 to 3, corresponding to the sapling, juvenile and adult stages, respectively, and Fisher’s C = 27.598 with p = 0.592 indicates that there is no significant difference between the constructed theoretical model and the observed data. The model fits well and is an acceptable one. The analysis revealed indirect effects of different life history stages on net photosynthetic rate. Specifically, as trees grow, the net photosynthetic rate of leaves tends to decrease gradually (Path coefficients = -0.378), which may be related to the resource allocation strategies of trees at different growth stages. Additionally, an increase in tree size has a significant direct negative effect on intercellular carbon dioxide concentration (Path coefficients = -0.486) and a significant direct positive effect on leaf dry matter content (Path coefficients = 0.218), indicating that tree growth has complex impacts on photosynthesis and nutrient storage. Overall, increases in leaf specific hydraulic conductivity (Path coefficients = 0.286), and nitrogen content (Path coefficients = 0.223) can significantly enhance the net photosynthetic rate of leaves. These enhancements in traits may help trees utilize water and nutrients more efficiently, thereby promoting photosynthesis (**Figure 5**).

### Coupling between net photosynthetic rate and hydraulic efficiency across life history stages

**Figure 6:**
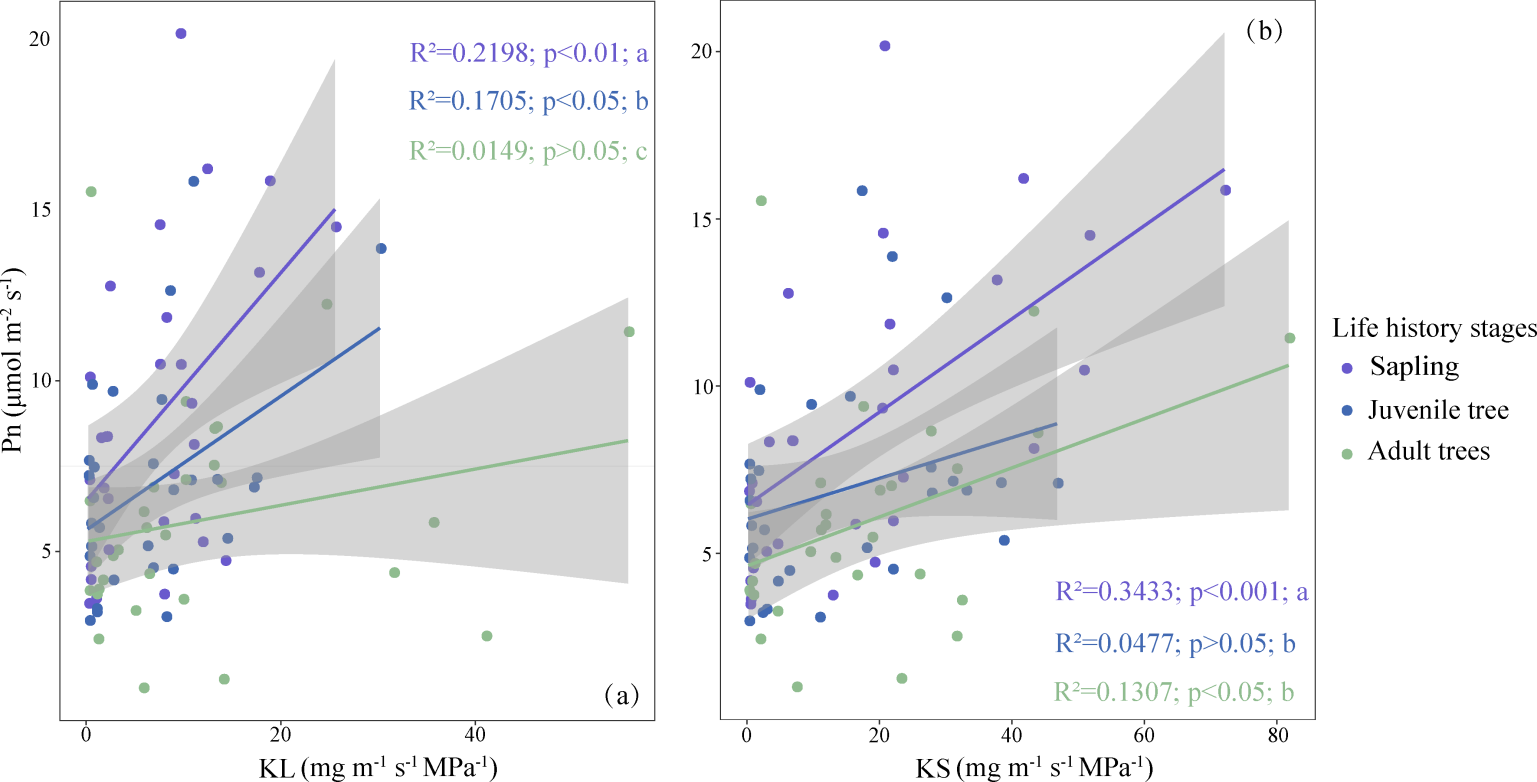
Coordination between net photosynthetic rate (Pn) and water use efficiency across life history stages. **(a)** Coordination between leaf specific hydraulic conductivity (KL) and net photosynthetic rate (Pn). **(b)** Coordination between sapwood specific hydraulic conductivity (KS) and net photosynthetic rate (Pn). Different lowercase letters denote significant differences in regression slopes among groups. Trait abbreviations are as defined in **Figure 1**.

In trees across different life history stages, the coordination between net photosynthetic rate and hydraulic efficiency remains largely consistent but with slight variations. Specifically, in the relationship between leaf specific hydraulic conductivity and net photosynthetic rate, saplings and juvenile trees exhibit a significant correlation, while this correlation is not significant in adult trees. In terms of the relationship between sapwood specific hydraulic conductivity and net photosynthetic rate, saplings and adult trees show a significant correlation, whereas this correlation is not significant for juvenile trees (**Figure 6**).

Trees at different life history stages display significant differences in the slope of the relationship between hydraulic efficiency and net photosynthetic rate. This indicates that during the growth process of trees, the extent to which net photosynthetic rate changes with hydraulic efficiency gradually decreases (**Figure 6**).

## Discussion

### The magnitude and distribution of trait variability

Our results indicate that hydraulic traits exhibit higher variability compared to chemometric traits, with a difference in the interquartile coefficient reaching an order of magnitude (**Figure 2A**). This finding is consistent with cross-species studies by Rosas et al. (2019), suggesting that water transport capacity is highly sensitive to microenvironmental changes. For example, temperature variations may prompt adjustments in xylem water transport capacity to meet plant transpiration demands, leading to differences in water transport capacity with environmental changes (Zhu and Zhao, 2023; Will et al., 2013; Pandey et al., 2018). On the other hand, the low variability of leaf chemometric traits indicates that these traits are more stable within small-scale environments. Global studies have established that leaf nitrogen (N) and phosphorus (P) concentrations generally decrease, while the leaf N:P ratio increases, with decreasing latitude (i.e., increasing mean annual temperature) (Wright et al., 2017; Hedin, 2004; Reich and Oleksyn, 2004). These trends reflect the stability of leaf chemometric traits on a global scale and the impact of environmental factors on these traits.

Trait variability is primarily distributed among species and within species, while trait variations between different life history stages are relatively minor (**Figure 2B**). Intraspecific trait variability accounts for approximately 40% of the total variability (**Figure 2B**), a finding that aligns with numerous studies. For instance, Ondej et al. (2019) found intraspecific trait variability to range between 30% and 60%, and in some species, the range of trait values is extremely broad. This indicates that within a species, trait values exhibit high variability, which depends not only on the traits themselves but also on the species. This implies that the behavior of individuals or species groups (including their responses to and impacts on the environment) is not fixed but may vary with changing environmental conditions (Albert et al., 2010). These intraspecific trait variations could help plants enhance their resource acquisition capabilities and gain competitive advantages (Wang et al., 2022).

Across different life history stages, saplings tend to exhibit higher physiological capacities such as hydraulic conductivity and net photosynthetic rate, while juvenile and adult trees show a greater tendency towards functional traits like higher leaf dry matter content, specific leaf area, and chlorophyll content index (**Figure 3**). These results indicate that as trees grow, there is a trend towards decreased physiological function. However, due to the better lighting conditions experienced by the leaves of taller trees, their chlorophyll content index is often higher than that of saplings. Additionally, due to increased evaporative demand and longer vertical water transport paths, they require stronger water storage capacity. Studies have shown that to mitigate the negative impacts of increased hydraulic pressure due to height, trees adapt their leaf characteristics to match their hydraulics. For example, through physiological adaptations (such as osmotic adjustment and cell wall thickening) to enhance the water storage capacity of leaves (Blackman et al., 2009; Maréchaux et al., 2015; Shiraki et al., 2017). Morphological adaptation measures (such as increased leaf thickness) can compensate for reduced hydraulic conductivity by preventing water loss from the leaf surface (Niinemets, 2010; Coble et al., 2014; Coble and Cavaleri, 2017). Thus, larger trees may exhibit higher leaf dry matter content and specific leaf area.

### Relative importance of different functional traits on net photosynthetic rate

Our results reveal that changes in traits such as stomatal conductance, transpiration rate, intercellular carbon dioxide concentration, and leaf specific hydraulic conductivity are important factors affecting the net photosynthetic rate. Notably, variables such as leaf specific hydraulic conductivity, sapwood specific hydraulic conductivity, and whole-branch hydraulic conductivity can explain 59.28% of the variation in net photosynthetic rate (**Figure 4**), indicating that leaf specific hydraulic conductivity plays a crucial role among all factors influencing the net photosynthetic rate. Leaves, as the primary organs for carbon and water exchange in plants (Jin and Wang, 2015), have an anatomical structure that determines the flow resistance of water passing through, which often constitutes a significant part of the plant’s overall hydraulic resistance (Sack et al., 2003; Grace et al., 2017). Considering that the hydraulic resistance in leaves accounts for a considerable proportion of the whole plant, the efficiency of delivering water to mesophyll cells may become a key factor limiting the net photosynthetic rate.

Leaf chemical traits and leaf photosynthetic traits explained 15.92% and 24.53% of the variation in net photosynthetic rate, respectively (**Figure 4**). This indicates that the impact of leaf carbon, nitrogen, and phosphorus content on the net photosynthetic rate is comparable to the importance of traits such as stomatal conductance, transpiration rate, and intercellular carbon dioxide concentration. Further studies have shown that increases in leaf specific hydraulic conductivity, stomatal conductance, and nitrogen content can significantly enhance the leaf’s net photosynthetic rate (**Figure 5**). Additionally, research has indicated that nitrogen and phosphorus are essential nutrients for photosynthesis, and the photosynthetic capacity of leaves is mainly limited by these two elements (Wang et al., 2018). The photosynthetic capacity of plants globally is generally constrained by the concentrations of nitrogen and phosphorus in the leaves (Wright et al., 2004; Maire et al., 2015), as photosynthetic capacity is typically positively correlated with the concentrations of these elements (Conroy et al., 1986; Guan and Wen, 2011; Kattge et al., 2009). Nitrogen or phosphorus deficiency reduces the net photosynthetic rate (Hikosaka, 2004). Thus, our findings emphasize the central role of leaf specific hydraulic conductivity in plant water transport and photosynthesis and reveal the significance of leaf chemical composition in regulating the net photosynthetic rate.

### Regulation of net photosynthetic efficiency in trees across different life history stages

Barnard and Ryan (2003) proposed three key factors explaining the deceleration of higher tree growth rates: first, stomata (along with associated transpiration and photosynthesis) must respond to changes in hydraulic resistance; second, hydraulic resistance inevitably increases with tree height or age; third, photosynthetic efficiency of leaves in older trees should decrease. Our study results show that as trees grow, both the net photosynthetic rate and hydraulic efficiency of leaves exhibit a declining trend, and their synergy also weakens, supporting these three elements (**Figure 6**). Additionally, for the same net photosynthetic rate, large trees have higher water demands than small trees, reflecting the trade-off between carbon assimilation and water loss. As trees grow taller, water loss becomes more severe to maintain the same amount of carbon assimilation. The ability of plant roots to absorb water and nutrients is limited, and these resources need to be transported throughout the plant to support its life activities. With increasing tree height, plants cannot continuously meet local growth requirements, thereby limiting tree growth. Extensive research points to a fact: the expansion space for plant root systems is limited, affecting the ability of plants to access soil resources, which impacts the growth status of the aboveground parts of the plant (Warren et al., 2015; Shanin et al., 2015);. However, whether it is the maximum value of fine root biomass underground that cannot provide sufficient water and nutrients leading to slower tree growth, or an upper limit on leaf area index at the stand level ultimately restricting the maximum carbon assimilation capacity at the stand level (Xu et al., 2012; Bowman et al., 2013), causing the fine root biomass underground to no longer increase and reach the maximum value due to spatial limitations, remains to be further studied. It is clear that in forest ecosystems, small trees cannot gain greater advantages in competition for sunlight, water, and other resources (Wawrzyniak et al., 2020), while large trees face higher water losses under the same carbon assimilation conditions and the challenge of supplying sufficient water to their vast bodies. Therefore, only when the size of trees is appropriate for their own survival strategies can they better survive in forest ecosystems.

## Conclusions

The variability in plant functional traits exhibits significant differences, with the coefficient of variation between the most labile and the most stable traits differing by an order of magnitude. These variations are primarily distributed among species and within species, while less variation is observed across different life histories. As trees grow, most traits show a declining trend; however, some traits may increase due to better light conditions or fulfilling specific physiological demands. An increase in leaf-specific hydraulic conductivity, stomatal conductance, and nitrogen content can significantly enhance the net photosynthetic rate of leaves, reflecting a close coordination between water transport and photosynthesis in plants. Since leaves are the primary organs for carbon and water exchange in plants, the efficiency of water delivery to mesophyll cells, carbon dioxide exchange through stomata, and leaf nitrogen concentration are crucial for the net photosynthetic rate. As trees grow, both the net photosynthetic rate and hydraulic efficiency of leaves decline, and their synergy weakens. This implies that larger trees face higher water loss while maintaining the same carbon uptake and need to supply sufficient water for their massive bodies, which gradually reduces height growth and ultimately limits tree growth.

## Data Availability

The original contributions presented in the study are included in the article/supplementary material. Further inquiries can be directed to the corresponding author.
